# Supplementary material for: Taxonomic analysis of metagenomic data with kASA
Source: Nucleic Acids Res. 2021 Mar 30;49(12):e68. doi: 10.1093/nar/gkab200 (PMC8266618; doi:10.1093/nar/gkab200)
Supplement: gkab200_Supplemental_Files [file gkab200_supplemental_files.zip › Suppl_D.zip!/OPAL_comparison_new/spider_plot.pdf]

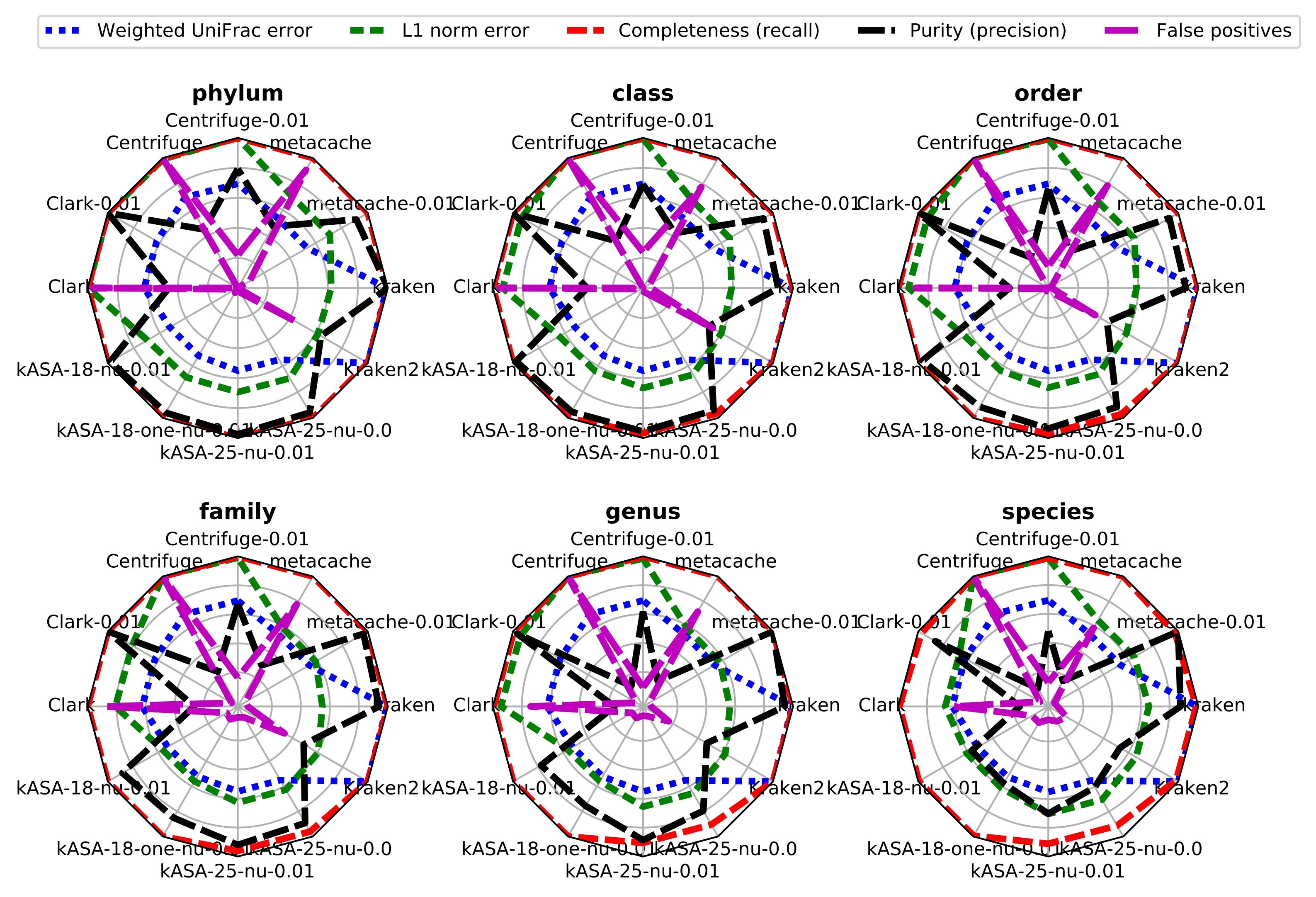

Weighted UniFrac error
L1 norm error
Completeness (recall)
Purity (precision)
False positives
phylum
class
order
family
genus
species
Centrifuge-0.01
Centrifuge
metacache
metacache-0.01
Clark-0.01
Clark
Kraken
Kraken2
kASA-18-nu-0.01
kASA-18-one-nu-0.01
kASA-25-nu-0.0
kASA-25-nu-0.01
